# Up-regulation of matrix metalloproteinases-9 in the kidneys of diabetic rats and the association with neutrophil gelatinase-associated lipocalin

**DOI:** 10.1186/s12882-021-02396-w

**Published:** 2021-06-03

**Authors:** Huayu Yang, Haiping Chen, Fenghua Liu, Qing Ma

**Affiliations:** 1 Division of Geriatrics, Medical and Health Care Center, Beijing Friendship Hospital, Capital Medical University, No. 95, Yong’an Road, Xicheng District, 100050 Beijing, People’s Republic of China; 2 Department of Nephrology, Emergency General Hospital, Beijing, People’s Republic of China

**Keywords:** Diabetic rats, Matrix Metalloproteinases-9, Neutrophil Gelatinase-associated Lipocalin

## Abstract

**Background:**

Matrix metalloproteinases-9 (MMP-9) can regulate extracellular matrix deposition in diabetic glomerular injury. However, it remains unknown whether MMP-9 is involved in the renal tubular injury. Meanwhile, neutrophil gelatinase-associated lipocalin (NGAL), defined as a biomarker of proximal tubular injury, may influence MMP-9 by forming the MMP-9/NGAL complex. The aim of this study was to investigate MMP-9 expression in proximal renal tubules and the relationship of MMP-9 and NGAL in diabetic rat model treated with Valsartan.

**Methods:**

Sprague Dawley rats were randomly divided into three groups: Diabetic group, Control group, and Treated group. The diabetic rat model was established by injection of streptozotocin. Related indexes were measured at the end of the 2nd, 4th, 8th and 12th week post-modeling.

**Results:**

In diabetic groups, the concentrations of MMP-9 markedly increased in the serum and urine of rats in the early stage, even before the appearance of pathological albuminuria. Markedly elevated MMP-9/NGAL complex concentrations were also tested in diabetic groups. Western blot and qPCR tests confirmed that MMP-9 expression levels in the proximal renal tubular epithelial cells of diabetic rats were significantly higher than in control groups (*P* < 0.05). Correlation analysis showed that MMP-9 was positively correlated with NGAL at both protein and gene expression levels. In addition, Valsartan observably reduced tubular injury as well as MMP-9 expression in diabetic rats.

**Conclusions:**

In diabetic kidney injury, the expression of MMP-9 in the proximal renal tubular epithelial cells was significantly increased. Besides, a positive correlation was found between MMP-9 and NGAL expression, along with high levels of MMP-9/NGAL complex, which indicated that NGAL might participate in the regulation of MMP-9 expression. The administration of Valsartan may reduce this effect.

## Background

Tubular damage has a pivotal role in the progression of diabetic nephropathy (DN). Our previous study discovered that proximal tubular injury appears before the appearance of pathological proteinuria in the early stage of DN, thus suggesting that tubular lesions occur earlier than glomerular lesions [1]. With the evolvement of DN, the tubular lesions severely aggravate the decline of renal function.

Matrix metalloproteinases (MMPs), belonging to a family of Zn^2+^- dependent and Ca^2+^-dependent endopeptidases, are abnormally expressed in various kidney diseases. Previous studies have confirmed that matrix metalloproteinase-9 (MMP-9) can regulate extracellular matrix (ECM) degradation during renal fibrosis [2, 3]. In addition, high expression of MMP-9 can induce epithelial-mesenchymal transformation (EMT) in tubular cells, which may be another mechanism for inducing renal fibrosis [4, 5]. Mice lacking matrix MMP-9 gene have been shown to have reduced disruption of the tubular basement membrane and expression of fibronectin, as well as deposition of total tissue collagen [6]. Contrary, elevated expression of MMP-9 in DM has been shown to directly stimulate the secretion of activated growth factors, such as TNF-α、IL-1β and VEGF, leading to renal injury [7, 8].

Previous studies have found abnormally expressed MMP-9 located in integral renal tissues or glomerulus in a hyperglycemic environment. However, the exact role of MMP-9 expression and activity in the diabetic kidney remains unclear.

Multiple mechanisms may be involved in the regulation of MMP-9 expression in DN. In most cells, MMP-9 enzyme activity can be regulated by some endogenous metalloprotease inhibitors known as tissue inhibitors of metalloproteinases (TIMPs). Recent reports have shown that MMP-9 activities can also be influenced by neutrophil gelatinase-associated lipocalin (NGAL). Our previous study suggested NGAL as a specific biomarker for diabetic tubular injury [1]. Some studies suggested that a formation of an MMP-9/NGAL complex can prevent MMP-9 degradation and prolong its activity [5, 9, 10]. Moreover, several clinical studies have investigated the associations of MMP-9/NGAL complex and diabetic renal injury. However, whether this binding effect exists in diabetic kidney tissue is still not well understood.

Pre-clinical studies suggested that the progression of DN was related to persistent activation of the renin-angiotensin system. The standard drug treatment for the management of kidney injury involves the administration of angiotensin-converting enzyme inhibitors or angiotensin II type 1 receptor blockers (ARBs) [11, 12]. However, the detailed mechanisms of ARBs in protecting the kidneys from DN damage are not fully understood.

In this study, we investigated the MMP-9 expression in proximal renal tubules and the relationship of MMP-9 and NGAL in diabetic models, as well as the effect of Valsartan on the MMP-9 expression.

## Materials and methods

### Experimental diabetic model

A total of 96 male adult Sprague Dawley rats (Vital River Laboratories, Beijing, China), weighting 180 -200 g were housed in an environment with a temperature of 22 ± 1 ºC, a relative humidity of 50 ± 1 %, and a light/dark cycle of 12/12 hr and fed with food and water *at libitum*. All animal studies (including the mice euthanasia procedure) were done in compliance with the regulations and guidelines of Capital Medical University (Beijing, China) institutional animal care and conducted according to the AAALAC and the IACUC guidelines.

Animals were randomly divided into three groups (32 rats per group): non-diabetic control group (Group N), diabetic group (Group D), and Valsartan treated group (Group T). The diabetic model was induced by injection of STZ (60 mg/kg of body weight, Sigma, St. Louis, MO, USA) dissolved in 0.1 mol/l citrate buffer (pH 4.5). Rats in the Valsartan treated group were gavaged with Valsartan (10 mg•kg^− 1^•d^− 1^ other two groups were treated with normal saline. After 72 h of STZ-injection, the blood glucose level was measured levels higher than 300 mg/dl indicated that the model was successfully constructed.

### Collection of blood and urine specimens

Urine samples were stored in a metabolic cage for 24 h at the end of 2nd, 4th, 8th, and 12th week after treatment was initiated. Eight rats in each group were euthanized using 10 % chloral hydrate 450 mg/kg injected into the abdominal cavity after each time point. Then, blood samples were taken from the heart. After centrifugation, the supernatant was retained for the corresponding detection. All urine and blood samples were stored at -80 ℃ and used for the study within 3 months after collection.

### Biochemical parameters tests

Serum creatinine (Scr) and blood urea nitrogen (BUN) were determined respectively at the end of the 2nd, 4th, 8^th,^ and 12th weeks. Urinary albumin was determined using ELISA kits (Groundwork Biotechnology Diagnostics, San Diego, CA, USA) at the same time as above. Then, the urinary albumin excretion rate (UAER) was calculated. Body weight and kidney weight of each rat were simultaneously measured in chronological order, followed by calculating the ratio of kidney weight and body weight.

### ELISA assay

The serum MMP-9 (sMMP-9), urinary MMP-9 (uMMP-9), serum NGAL (sNGAL), urinary NGAL (uNGAL), serum MMP-9/NGAL complex and urinary MMP-9/NGAL complex levels were measured using ELISA kits (Groundwork Biotechnology Diagnostics, San Diego, CA, USA).

### Western blot

Proteins extracted from renal medulla were separated on 12 % SDS–polyacrylamide gels and transferred to polyvinylidene difluoride membranes. After blocking with 5 % skim milk, the membranes were incubated overnight at 4 °C with primary antibodies against MMP-9 (goat polyclonal, 1:500, Santa Cruz, CA, USA) or NGAL (rabbit polyclonal, 1:5:00, Santa Cruz, CA, USA) or TIMP-1 (rabbit polyclonal, 1:5:00, Santa Cruz, CA, USA), followed by washing and incubation with secondary rabbit anti-goat IgG (1: 20,000, Santa Cruz, CA, USA) or goat anti-rabbit IgG (1: 20,000, Santa Cruz, CA, USA). The density of MMP-9, NGAL, and TIMP-1 staining were analyzed with Quantity One software.

### Real-time PCR

RNA was isolated with RNA Kit (Sunbio, Beijing, China). The cDNA was conducted with a Reverse Transcription Kit (Promega M170A). Real-time PCR was performed using Bio Easy SYBR Green I Real-Time PCR Kit (Sunbio, Beijing, China) with the following sequences of primers: MMP-9 forward: 5’- GCGCCGTGGTCCCCACTTAC − 3’; MMP-9 reverse: 5’-GCCGTCTCCGTTGCCATGCT-3’; NGAL forward: 5’-TGAACTGAAGGAGCGATTCG-3’; NGAL reverse: 5’-ATTGGTCGGTGGGAACAGA-3’; TIMP-1 forward: 5’- TAAAGCCTGTAGCTGTGCCC-3’; TIMP-1 reverse: 5’- AGCGTCGAATCCTTTGAGCA − 3’; GAPDH forward: 5’- ACCACCCAGCCCAGCAAGGAT-3’; GAPDH reverse: 5’- GGGGCTGAGTTGGGATGGGGAC-3’. The relative quantities were assessed by the comparative Ct method (2 − ΔΔCt).

### Immunohistochemical analysis of MMP-9

Paraffin-embedded kidney tissue slices (3 mm thickness) were deparaffinized. Then, the slices were treated with 3 % hydrogen peroxide in methanol for 10 min. Antigen retrieval was performed with citrate buffer for 5 min in a 100 °C water bath. The slices were then incubated with 10 % goat serum for 10 min at room temperature, followed by incubation with anti-MMP-9 antibody (1:500, Santa Cruz, CA, USA) at 4 °C overnight. Next, the slices were incubated with horseradish peroxidase for another 30 min. After incubation with secondary antibodies, the sites of peroxidase activity were carried out by 3, 3-diamino-benzidine tetrahydrochloride stain as a substrate, followed by hematoxylin staining. Every slide was examined MMP-9 stains in the normal and atrophic renal tubules.

### Statistical analysis

All data were expressed as means ± standard deviation (SD). Statistical analysis was performed with SPSS 19.0. Differences in variables were tested with one-way analysis of variance (ANOVA), followed by Bonferroni posthoc analysis. The Pearson correlation coefficient was performed to examine the correlations between variables. The difference was considered statistically significant when the *P*-value was < 0.05.

## Results

### Clinical parameters

Table 1 shows the basic characteristics of all the rats. Diabetic rats showed a gradual increase in blood glucose, Scr, and BUN compared to a non-diabetic control group in the same period. In addition, STZ-induced diabetic rats had an obvious increase in UAER. At the end of 2nd week, there was no significant difference (*P* > 0.05) in UAER in the Valsartan-treated rats and diabetic groups; yet, the levels decreased in week 4 compared to those in diabetic rats (*p* < 0.05) (Table 1).

**Table 1:** The characteristics of streptozotocin-induced diabetic rats at all stages

Week	Group	Scr (umol/l )	BUN (mmol/l)	UAER (ug/min)	Blood glucose
Week 2	N	46.00±7.80	7.23±1.61	0.98±0.51	7.18±1.98
	D	51.17±7.81*	12.42±3.37*	2.99±1.12*	25.93±1.49*
	T	52.63±8.21	13.05±2.65	3.10±0.62*	21.26±0.51*
Week 4	N	50.33±7.15	7.10±0.84	0.76±0.30	8.92±1.64
	D	57.43±4.86*	15.25±2.87*	4.91±0.12*	29.15±1.86*
	T	54.13±3.21	11.83±1.49*^#^	4.64±0.20*^#^	24.67±1.90*
Week 8	N	55.00±3.58	7.88±0.66	0.76±0.41	8.06±2.05
	D	63.50±5.75*	20.10±3.64*	5.91±0.15*	28.22±2.22*
	T	60.13±9.85	18.56±8.33*^#^	5.48±0.53*^#^	25.79±1.83*
Week 12	N	50.38±3.89	7.00±0.53	0.64±0.22	8.37±1.69
	D	64.88±7.59*	21.32±5.65*	6.79±0.32*	30.75±1.81*
	T	62.38±5.01*	14.76±2.37*^#^	4.71±0.25*^#^	26.85±0.97*

Mean±SD data were from non-diabetic control groups (N), diabetic groups (D), Valsartan treated group (T)

*P < 0.01 vs. non-diabetic control group in the same period

^**#**^*P* < 0.01 vs. diabetic group in the same period

### Detection of serum and urine indicators

**Fig. 1:**
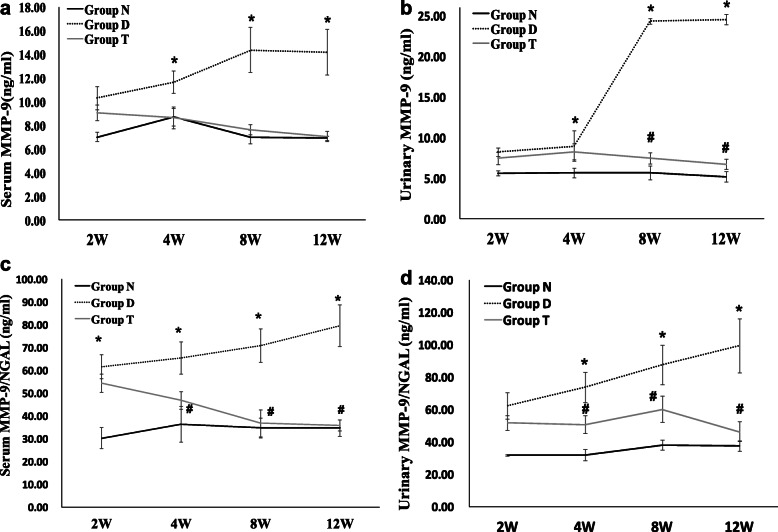
MMP-9 and MMP-9/NGAL complex levels in serum and urine among three groups. Data presented as serum MMP-9 (**a**), urinary MMP-9 (**b**), serum MMP-9/NGAL complex (**c**), and urinary MMP-9/NGAL complex (**d**) levels at 2nd, 4th, 8^th,^ and 12th weeks after streptozotocin injection. Groups presented as non-diabetic control groups (Group N), diabetic groups (Group D), and Valsartan treated groups (Group T). *P < 0.05 vs. non-diabetic control group in the same week; ^#^*P* < 0.05 vs. diabetic group in the same week

We detected MMP-9 concentration in blood and urine. As shown in Fig. 1a and b, the serum and urinary MMP-9 in diabetic rats gradually increased after week 2 compared with non-diabetic controls. In rats treated with Valsartan, serum MMP-9 and urinary MMP-9 concentrations decreased by about 1.4 and 3.8 times, respectively at the end of the 12th week. The changes in NGAL concentration in serum and urine were similar to those of MMP-9 (Fig. 1c and d). Serum MMP-9/NGAL complex and urinary MMP-9/NGAL complex concentrations were significantly higher in the diabetic group than in the non-diabetic control group in the same period. Similarly, the levels of the above indicators significantly decreased after treatment of Valsartan (Fig. 1e and f).

### Protein expression of MMP-9, NGAL, and correlation analysis

**Fig. 2:**
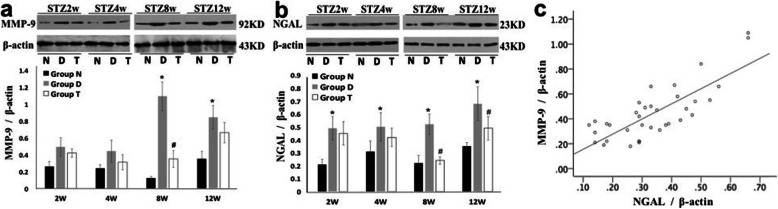
MMP-9 and NGAL protein expression in the renal medulla of diabetic rats. **a, b** The protein expression and semi-quantitative results of MMP-9 and NGAL. Three groups showed as non-diabetic control groups (Group N), diabetic groups (Group D), and Valsartan treated groups (Group T© A positive correlation of MMP-9 and NGAL protein expression. *P < 0.05 vs. non-diabetic control group in the same week; ^#^*P* < 0.05 vs. diabetic group in the same week

Western blot analysis revealed the expression of MMP-9 (92KD) and NGAL (23KD) protein in renal medulla extracts. Diabetes was associated with an increase in MMP-9 and NGAL expression (Fig. 2a and b). At the 12th week, MMP-9 and NGAL protein expression levels in diabetic rats respectively increased by 2.71-fold and 1.94-fold compared to the non-diabetic control group. As shown in Fig. 2a, b and a simultaneous decrease of MMP-9 and NGAL expression was prevented by Valsartan. Correlation analysis of MMP-9 and NGAL protein expression in Fig. 2c yielded a statistically-significant correlation of 0.78 (*p* < 0.05).

### Gene expression of MMP-9, NGAL, and correlation analysis

**Fig. 3:**
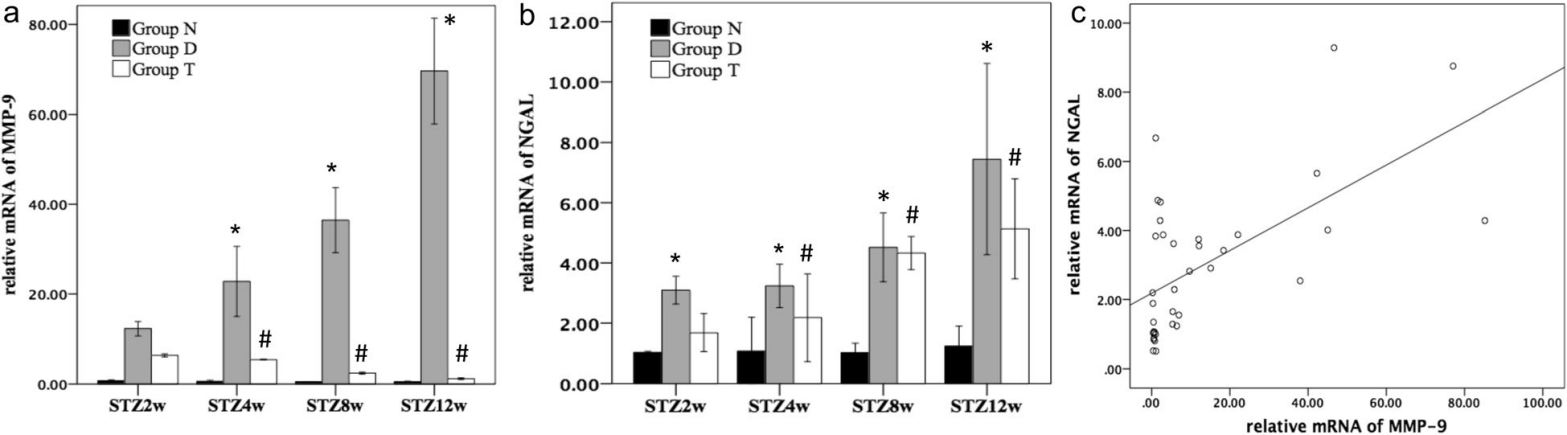
MMP-9 and NGAL mRNA expression in rats’ renal medulla in group N (non-diabetic control), group D (Streptozotocin-induced diabetic rats), and group T (Valsartan 10 mg•kg^− 1^•d^− 1^ treated) rats. **a, b** Relative MMP-9 mRNA and NGAL mRNA expression in Groups N, D, and T, normalized against that of glyceraldehyde-3-phosphate dehydrogenase (GAPDH). **c** A positive correlation of MMP-9 and NGAL gene expression. Data are the mean ± SD (*n* = 8 in each group). *P < 0.05 compared with Group N; ^#^*P* < 0.05 compared with Group D

Next, Real-time PCR was used to examine the expression of MMP-9 mRNA. Since the end of 2nd week, the mRNA expression for both NGAL (Fig. 3a) and MMP-9 (Fig. 3b) was elevated (*P* < 0.001) compared with non-diabetic controls (Fig. 3a and b, respectively). After treatment with Valsartan, gene expression of MMP-9 was significantly decreased at the end of 4th week, while gene expression of NGAL showed an obvious decrease at the end of 12th week (Fig. 1a and b, respectively). Correlation analysis of MMP-9 and NGAL gene expression in Fig. 3c yielded a statistically-significant correlation of 0.61 (*p* < 0.05).

### Histological examinations

**Fig. 4:**
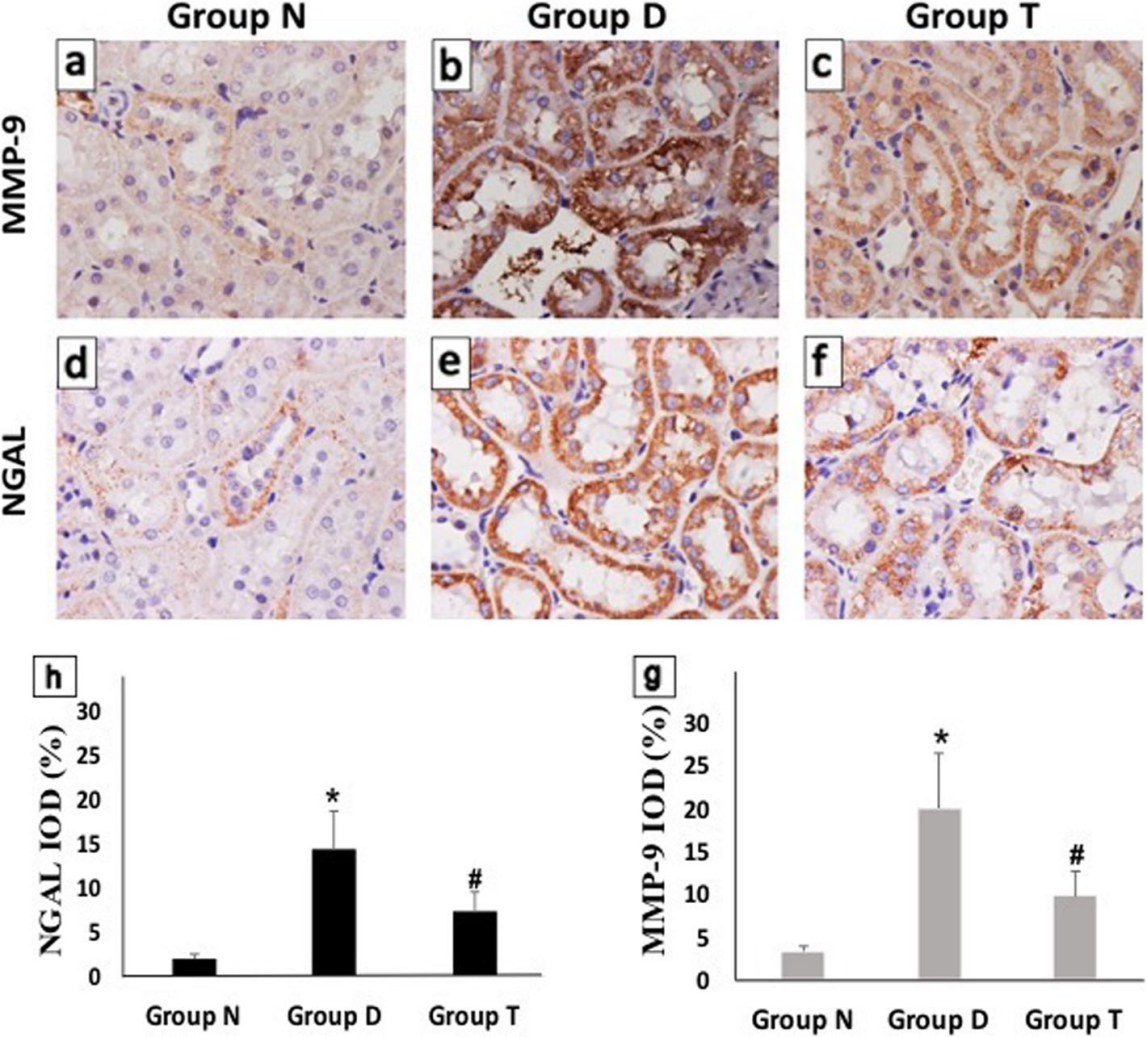
Immunohistochemical stain of epithelial cells of proximal renal tubules at the 12th week in each group of rats at magnifications 1,200x under a microscope. **a, d** Non-diabetic control group (Group N); **b, e** diabetic group (Group D); **c, f** Valsartan treated group (Group T). **g, h** Semi-quantitative analysis of MMP-9 (**g**) and NGAL (**h**). Data were expressed as %. *P < 0.05 compared with Group N; ^#^*P* < 0.05 compared with Group D

MMP-9 and NGAL expression in the proximal tubular epithelial cells at the 12th week of the study were examined by immunohistochemical staining and semi-quantitative analysis. Compared to a non-diabetic group, strong staining of MMP-9 and NGAL were found in the diabetic group. Contrary, the MMP-9, and NGAL staining were decreased in the Valsartan treatment group compared to the diabetic group. Consistently, semi-quantitative analysis revealed significant differences between every two groups (*P* < 0.05, shown in Fig. 4).

## Discussion

At the moment, available data on the expression of MMP-9 in diabetic kidneys remain conflicting. Szu-yuan et al. reported that MMP-9 was significantly increased in the glomeruli of diabetic mice. The same author reported that MMP-9 deficiency could attenuate diabetic renal injury by modulating podocyte functions and dedifferentiation [13]. However, other studies found a decrease in MMP-9 expression in diabetic rat renal tissue [14–16]. Yet, the expression of MMP-9 on renal tubular epithelial cells in diabetes mellitus remains unclear. In our study, elevated MMP-9 expression was observed in serum, urine, and renal tissues of diabetic rats. The follow-up observation showed a continuation of an uptrend with the aggravation of the renal injury. Furthermore, diabetic rats exhibited increased MMP-9 staining in proximal renal tubular epithelial cells. This inconsistent result may be attributed to the location where MMP-9 is expressed. It has been confirmed that in mice models, MMP-9 was mainly expressed in collecting duct cells and then in proximal tubule epithelial cells and podocytes [17]. Different expression sites were observed in the above studies, resulting in different observation results. Chen et al. [18] have shown that endocytosis of albumin could stimulate MMP-9 secretion by renal tubule epithelial cells under diabetic conditions in vitro, which is consistent with our data. In addition, some researchers suggested biphasic modulation of MMP-9 in the development of diabetic kidney damage [19]. Future studies are needed to further address these issues.

The specific mechanism of MMP-9 involved in the development of diabetic nephropathy is not fully understood. Existing studies have mainly focused on the regulation of MMP-9 on the accumulation of ECM in the glomerulus of diabetic rats, assuming that the abnormal expression of MMP-9 could lead to aberrant ECM degradation and accumulation. In our study, the expression level of MMP-9 in renal tubular epithelial cells in the diabetic group gradually increased with the disease’s progression, suggesting that MMP-9 may be involved in the pathogenesis of diabetic renal injury through other mechanisms. Renal fibrosis has been proved to be one of the crucial pathogenesis of DM, including glomerulosclerosis, interstitial fibrosis, tubular atrophy, and other manifestations. Recently, it has been reported that dysregulated MMP-9 may relate to the pathogenesis of renal fibrosis. Tsai et al. have noted that an increased MMP-9 expression in human atrophic tubular nuclear was associated with a greater interstitial fibrosis score (*r* = 0.40, *p* = 0.002), thus suggesting it may have a role in the process of renal injury or fibrosis [20]. Furthermore, Tan et al. have proved a pro-fibrotic role of MMP-9 in tubular cell EMT, and confirmed the pathogenesis of MMP-9 involvement in renal fibrosis through recruitment of osteopontin cleavage [4]. Similarly, Ling et al. reported that abnormal expression of MMP-9 was correlated with EMT in podocytes of diabetic rats [21]. More in-depth studies are needed to further explore the pathogenesis of MMP-9 in the process of diabetic kidney injury.

A previous study found that NGAL, a protein that is recognized as a marker of diabetic tubular injury, is abundantly present in diabetic rats [1], which is consistent with our findings. NGAL can form the MMP-9/NGAL complex by binding to MMP-9 [22, 23]. The biological activity of MMP-9 could be prolonged by combination with NGAL through preventing its degradation. In our study, the MMP-9/NGAL complex displayed increased expression in the serum and urine of diabetic rats. In addition, we further investigated the association between NGAL and MMP-9 gene and protein expression. A positive correlation was found between the two variables at both the gene and protein levels. NGAL’s ability to form this complex seems to be less specific since it has been observed in the serum of patients with malignant tumors [24].

Previous studies reported that Valsartan, which belongs to a kind of angiotensin II receptor blocker (ARB), has renoprotective effects in DN [25, 26]. However, limited evidence exists on the effect of Valsartan on MMP-9 expression in diabetes. In our research, diabetic rats exhibited significantly increased levels of serum and urinary MMP-9, NGAL, and MMP-9/NGAL complex. An increase in MMP-9 expression was prevented by Valsartan treatment 4 weeks after injection of STZ. Further experiments revealed a marked decrease of MMP-9 expression in the treatment group compared with a diabetic group from week 4. Besides, histologically, Valsartan obviously reduced renal proximal tubular injury at the 12th week of the study. The above results suggest that Valsartan could inhibit MMP-9 expression and alleviate the damage of renal tubular damage in STZ-induced diabetic rats. Interestingly, another experiment conducted in diabetic rats showed that Valsartan could block the up-regulation of TIMP-1, a natural inhibitor of MMP-9, thus preventing the progress of renal interstitial fibrosis [27]. Previous studies have shown that in the process of renal fibrosis, the expression of MMP-9 and TIMP-1 were both increased, which resulted in an imbalanced/decreased MMP-9/TIMP-1 ratio and led to reduced capacity of MMP-9 to degrade ECM. Therefore, ARBs may be involved in the progression of diabetic renal fibrosis by regulating pro-fibrotic factors [28].

## Conclusions

The expression of MMP-9 was significantly increased in diabetic renal tubular epithelial cells. Besides, a positive correlation was found between MMP-9 and NGAL expression, along with high levels of MMP-9/NGAL complex. Valsartan were able to improve above alternations.

## Data Availability

The datasets used or analyzed during the current study are available from the corresponding author on reasonable request.

## Abbreviations

MMP-9: Matrix metalloproteinases-9
NGAL: Neutrophil gelatinase-associated lipocalin
DN: Diabetic nephropathy
MMPs: Matrix metalloproteinases
ECM: Extracellular matrix
EMT: Epithelial-mesenchymal transformation
TIMPs: Tissue inhibitors of metalloproteinases
Scr: Serum creatinine
BUN: Blood urea nitrogen
UAER: Urinary albumin excretion rate.
sMMP-9: Serum MMP-9
uMMP-9: Urinary MMP-9
sNGAL: Serum NGAL
uNGAL: Urinary NGAL
ARB: Angiotensin II receptor blocker
